# The Role of Advanced Magnetic Resonance Imaging Sequences in Multiple Sclerosis

**DOI:** 10.7759/cureus.67759

**Published:** 2024-08-25

**Authors:** Muhammad I Siddiqui, Amanullah Khan, Kamran I Memon, Muhammad I Farid, Muhammad Kashif, Dureali Mirjat, Maryam Ahmad, Tauseef Raza, Muhammad H Amjad

**Affiliations:** 1 Department of Diagnostic Radiology, North West General Hospital and Research Center, Peshawar, PAK; 2 Department of Clinical Imaging, Sheikh Shakhbout Medical City, Abu Dhabi, ARE; 3 Department of Imaging, Cleveland Clinic, Abu Dhabi, ARE; 4 Department of Electrical and Computer Engineering, Air University, Islamabad, PAK; 5 Department of Medicine, Arizona College of Osteopathic Medicine, Midwestern University, Glendale, USA; 6 Department of Medicine and Surgery, Arizona College of Osteopathic Medicine, Midwestern University, Glendale, USA; 7 Department of Medicine and Surgery, Shalamar Medical and Dental College, Lahore, PAK; 8 Department of Orthopedics, Khyber Medical University (KMU) Institute of Medical Sciences, Kohat, PAK; 9 Department of Neurosurgery, Hameed Latif Hospital, Lahore, PAK

**Keywords:** functional magnetic resonance imaging, magnetization transfer imaging, diffusion tensor imaging, diffusion-weighted imaging, multiple sclerosis

## Abstract

Background

The neurological condition known as multiple sclerosis (MS) is crippling and has a complicated pathogenesis as well as a wide range of clinical symptoms, including fatigue, difficulty walking, numbness or tingling, muscle spasms and spasticity, weakness, vision problems, dizziness and vertigo, bladder and bowel dysfunction, cognitive impairment, and emotional changes. The complete scope of MS pathology cannot be fully captured by conventional magnetic resonance imaging (MRI) sequences, which has led to the investigation of sophisticated MRI methods for better diagnosis and treatment.

Objective

This study aims to evaluate the clinical relevance of advanced MRI sequences in multiple sclerosis.

Methodology

A retrospective cohort study was conducted across multiple specialized medical centers renowned for treating neurological disorders, particularly multiple sclerosis, and involved 310 patients with diverse geography seeking treatment throughout 2022. Records were searched to obtain patient information, demographics, and treatment history. Descriptive statistics and t-tests were among the statistical studies that investigated relationships between MRI biomarkers and clinical factors to help with the diagnosis and treatment of MS. A p-value of <0.05 was significant.

Results

The research group consisted of 310 MS patients, the majority of whom were female (67.42%) and had a mean age of 34.7 years. With hypertension (14.52%) and hyperlipidemia (19.35%) as prevalent comorbidities, the majority of patients (72.26%) were on disease-modifying treatments. The results of advanced MRI showed that lesions with white matter had higher mean diffusivity (1.25 ± 0.15 mm²/s) on DWI, lesions with reduced magnetization transfer ratio (MTR) (0.15 ± 0.03) on MTI, and lesions with reduced fractional anisotropy (FA) (0.40 ± 0.08) on diffusion tensor imaging (DTI). Additionally, the blood oxygen level-dependent (BOLD) signals in cognitive processing regions (0.75 ± 0.10) on functional MRI were different from those with normal-appearing white matter (0.40 ± 0.08).

Conclusion

Advanced MRI sequences are essential for bettering MS diagnosis, prognosis, and treatment because they link imaging biomarkers to important clinical parameters, which improves patient care and quality of life.

## Introduction

The central nervous system's nerve fibers become demyelinated in multiple sclerosis (MS), a complicated neurological illness that impairs several senses, motor skills, and cognitive abilities [1,2]. Millions of people worldwide suffer from this long-term autoimmune disease, which tends to strike young adults more often and frequently leaves them severely disabled and with a worse quality of life [3,4]. The exact cause of MS is still unknown after decades of study, which makes it difficult to diagnose the disease, predict its prognosis, and track its course of therapy. But improvements in medical imaging methods, especially magnetic resonance imaging (MRI), have completely changed how we see and treat this crippling illness [5].

Traditional MRI sequences, which identify localized lesions suggestive of demyelination and inflammation in the central nervous system, have long been essential in the diagnosis of multiple sclerosis [6,7]. These sequences include T1-weighted, T2-weighted, and fluid-attenuated inversion recovery. However, these conventional sequences could not fully represent the pathophysiology of MS, particularly in the early phases of the illness or in lesions that are clinically quiet [8]. As a result, using improved MRI sequences to improve sensitivity and specificity in MS diagnosis and treatment has gained popularity [9].

The use of diffusion-weighted imaging (DWI) and diffusion tensor imaging (DTI), which measure the transport of water molecules inside tissues and provide insights into microstructural alterations linked to MS pathophysiology, is one such innovation [10]. Even in the absence of obvious lesions seen on traditional MRI sequences, DWI and DTI may identify minute changes in tissue integrity, such as axonal injury and microstructural disruption [11]. Furthermore, quantitative metrics like mean diffusivity (MD) and fractional anisotropy (FA) that are generated from DWI and DTI have shown potential as biomarkers for MS therapy response and disease progression [12].

Magnetization transfer imaging (MTI) is another new method that assesses the exchange of magnetization between protons in free water and protons bound to macromolecules like myelin [13]. Through the measurement of the extent of magnetization transfer between these pools, MTI is able to distinguish minute variations in the integrity and content of myelin, offering important insights into the processes of demyelination and remyelination in MS lesions [14]. Furthermore, MTI may function as a proxy indicator of disease activity and therapy effectiveness, enabling customized treatment plans for MS patients [15].

The measurement of brain activity and connection changes linked to neuroplasticity and cognitive impairment has been made possible by the increasing use of functional magnetic resonance imaging (fMRI) in the study of MS [16]. fMRI provides insights into adaptive processes and compensatory networks by elucidating functional rearrangement within the CNS in response to MS-related damage via the measurement of blood oxygen level-dependent (BOLD) signals. Additionally, by identifying indicators of cognitive reserve and disease progression in MS patients, fMRI may help with prognostication and therapy selection [17, 18].

Research objective

This study aims to evaluate the clinical relevance of advanced MRI sequences in multiple sclerosis.

## Materials and methods

Study design and settings

This retrospective cohort research was carried out at the District Head Quarter Teaching Hospital Kohat, Hayatabad Medical Complex, Hameed Latif Hospital, and Sheikh Shakhbout Medical City, which are well-known for their proficiency in treating neurological conditions such as multiple sclerosis. The research included a wide range of patients from different hospitals seeking treatment for multiple sclerosis over the course of a year, from January 2022 to December 2022.

Inclusion and exclusion criteria

Ages 18 and older, male or female, diagnosed with MS, and having completed advanced MRI sequences (such as DWI, DTI, MTI, and fMRI) to evaluate MS pathology are the inclusion criteria. The study includes patients with a variety of clinical characteristics and demographic backgrounds who are either receiving or not receiving DMTs and who are with or without typical MS comorbidities. Exclusion criteria include inability to undergo an MRI, neurological conditions, incomplete medical records, significant motion artifacts, history of major neurological interventions, major neurological interventions previously undergone, or acute exacerbations of MS requiring immediate medical attention.

Sample size

The predicted frequency of MS in the area and the expected effect size needed to get sufficient statistical power were used to establish the sample size, which came out to be 310 patients. It was determined that this sample size would be enough to identify clinically significant correlations between advanced MRI results and several clinical outcomes pertaining to MS diagnosis, prognosis, and treatment.

Data collection

Comprehensive patient data was taken out of electronic medical records, including demographics, clinical features, length, severity, and treatment history. Specialist radiologists thoroughly examined advanced MRI sequences, such as DWI, DTI, MTI, and fMRI, to evaluate their significance in the diagnosis, prognosis, and treatment of MS.

MRI protocol

MRI scans were performed using a 3T scanner at participating hospitals, incorporating advanced sequences including Diffusion-Weighted Imaging (DWI) with b-values of 0 and 1000 s/mm², Diffusion tensor imaging (DTI) with a 30-direction gradient scheme, magnetization transfer imaging (MTI) for magnetization transfer ratio (MTR), and fFunctional MRI (fMRI). These sequences were optimized for high resolution and signal-to-noise ratio to accurately evaluate MS pathology.

Statistical analysis

Descriptive statistics were conducted in SPSS version 23.0 (IBM Inc., Armonk, New York) to analyze the clinical and demographic profiles of the cohort. Parametric t-tests were utilized to compare means between groups, while non-parametric Mann-Whitney U tests were applied when data assumptions weren't met. Correlation analyses, employing Pearson's or Spearman's coefficients, explored relationships between MRI findings and clinical parameters. A significance threshold of p<0.05 was set to evaluate correlations. 

Ethical approval

The study was approved by the Ethical Committee of District Head Quarter Teaching Hospital, Kohat Development Authority, Kohat (195/K-21). Informed permission was not required due to the retrospective nature and use of de-identified patient data, minimizing biases and safeguarding patient privacy.

## Results

The clinical characteristics and demographics of MS patients are shown in Table 1. With a standard deviation of 9.2 years and a mean age of 34.7 years, the research included 310 patients. The patients' ages range from 18 to 30 years old (n=110, 35.48%), 31 to 40 years old (n=120, 38.72%), 41 to 50 years old (n=60, 19.35%), and above 50 years old (n=20, 6.45%). Of the sample, 209 patients (67.42%) are female, and 101 patients (32.58%) are male. Fifty (16.13%) have completed their elementary education, 180 (58.06%) have completed their secondary education, and 80 (25.81%) have completed their higher education. The illness lasts for 6.5 years on average. There are three categories for disease severity: mild (n=98, 31.61%), moderate (n=137, 44.19%), and severe (n=75, 24.19%) symptoms. The majority of patients (n=224, 72.26%) are undergoing disease-modifying treatments (DMTs). Hypertension (n=45, 14.52%) and hyperlipidemia (n=60, 19.35%) are common comorbidities. In terms of employment, there are 150 (48.39%) working people, 90 (29.03%) jobless people, and 70 (22.58%) retired people.

**Table 1:** Demographic and clinical characteristics of patients with multiple sclerosis

Characteristic	Number of patients (n)	Percentage
Age (years)	34.7 ± 9.2 (mean ± SD)	
Age group		
18-30 years	110	35.48
31-40 years	120	38.72
41-50 years	60	19.35
Above 50 years	20	6.45
Gender		
Female	209	67.42
Male	101	32.58
Education level		
Primary	50	16.13
Secondary	180	58.06
Tertiary	80	25.81
Disease duration (years)	6.5 ± 3.1 (Mean ± SD)	
Disease severity		
Mild	98	31.61
Moderate	137	44.19
Severe	75	24.19
Disease-modifying therapies (DMTs)		
Yes	224	72.26
No	86	27.74
Comorbidities		
Hypertension	45	14.52
Diabetes	30	9.68
Hyperlipidemia	60	19.35
Depression	35	11.29
Employment status		
Employed	150	48.39
Unemployed	90	29.03
Retired	70	22.58

Multiple patchy foci of T2 and fluid-attenuated inversion recovery (FLAIR) hyperintensity are observed in the cerebral white matter, with involvement of the periventricular region. One lesion in the right periventricular region shows restricted diffusion. Advanced MRI metrics include higher mean diffusivity (MD) in white matter lesions, lower fractional anisotropy (FA) in seemingly normal white matter, reduced magnetization transfer ratio (MTR) in lesions, and altered blood oxygen level-dependent (BOLD) signals in cognitive processing areas.

**Figure 1:**
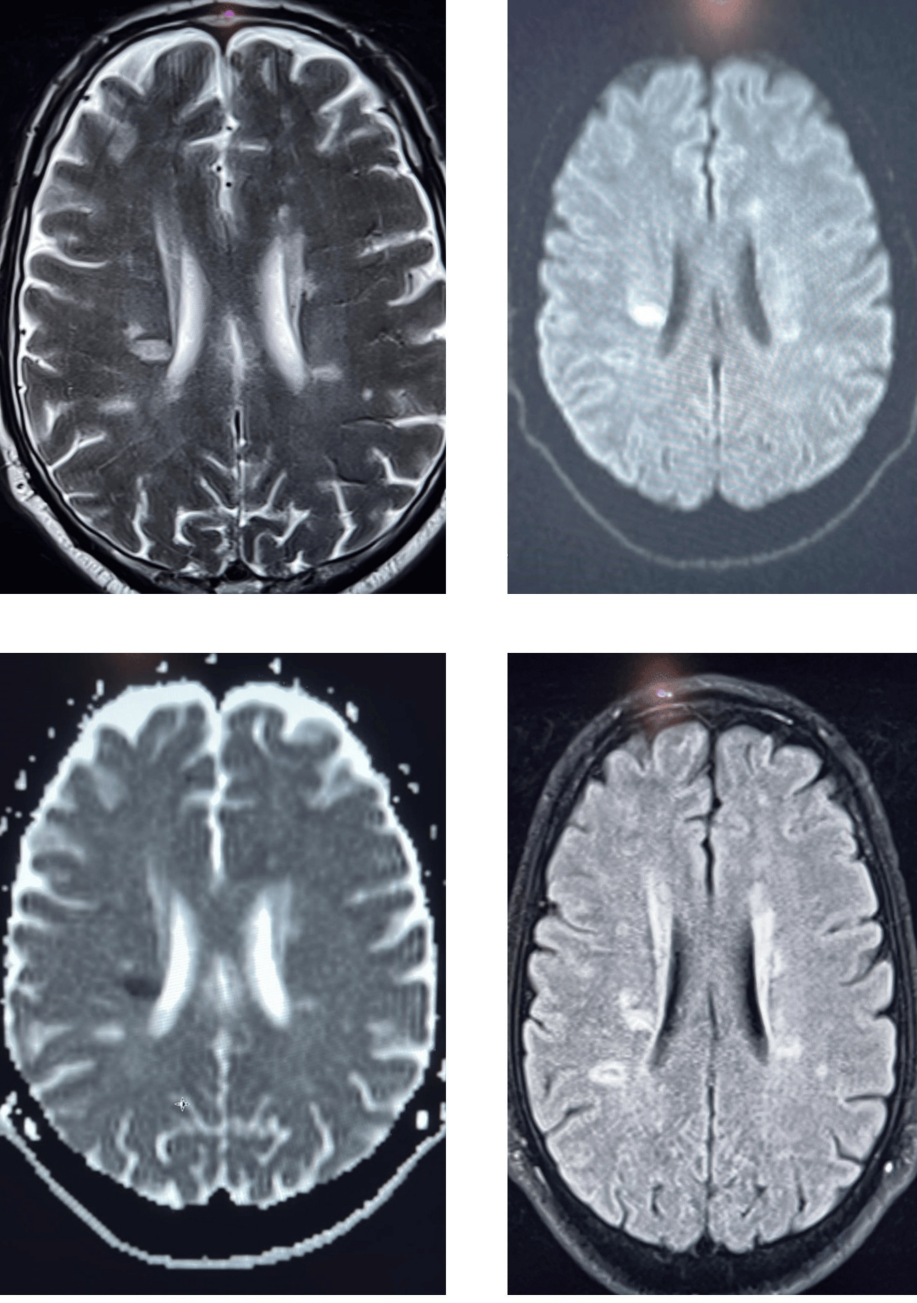
MRI findings in multiple sclerosis

Advanced MRI findings in MS patients are summarized in Table 2. DWI indicates a higher mean diffusivity (MD) in white matter lesions, ranging from 1.05 to 1.45 mm²/s in 310 individuals, with a mean value of 1.25 ± 0.15 mm²/s. Within the same sample size, DTI shows lower FA in white matter that seems normal, with a mean value of 0.40 ± 0.08 and a range of 0.30 to 0.50. In lesions, MTI reveals a lower magnetization transfer ratio (MTR), with a range of 0.10 to 0.20 and a mean value of 0.15 ± 0.03. BOLD signals in cognitive processing areas are shown to be changed in fMRI; the mean value is 0.75 ± 0.10, with a range of 0.65 to 0.85.

**Table 2:** Summary of advanced MRI findings in patients with multiple sclerosis DWI - diffusion-weighted imaging; DTI - diffusion tensor imaging; MTI - magnetization transfer imaging; fMRI - functional magnetic resonance imaging; BOLD - blood oxygen level-dependent

MRI modality	Key findings	Mean ± SD	Range
DWI	Increased mean diffusivity in white matter lesions	1.25 ± 0.15 mm²/s	1.05 - 1.45 mm²/s
DTI	Reduced fractional anisotropy in normal-appearing white matter	0.40 ± 0.08	0.30 - 0.50
MTI	Decreased magnetization transfer ratio in lesions	0.15 ± 0.03	0.10 - 0.20
fMRI	Altered BOLD signals in cognitive processing regions	0.75 ± 0.10	0.65 - 0.85

The relationship between DWI results and clinical indicators in MS patients is shown in Table 3. The corresponding p-values, which show the significance of the observed correlations, are given with the mean diffusivity (MD) values in the lesions. The findings reveal that lesion load also correlates with MD values (mean MD=0.91 ± 0.08 mm²/s, p=0.003) and that an increased MD in lesions correlates strongly with disease severity (mean MD=0.85 ± 0.12 mm²/s, p<0.001). Furthermore, there is a correlation between cognitive performance and changes in MD (mean MD=0.88 ± 0.09 mm²/s, p=0.012), and treatment response is predicted by changes in MD (mean MD=0.83 ± 0.11 mm²/s, p=0.025). These results highlight the therapeutic usefulness of DWI in assessing MS patients' lesion load, cognitive function, disease severity, and responsiveness to therapy.

**Table 3:** Association between DWI findings and clinical parameters P-value of <0.05 is considered significant MD - mean diffusivity; DWI - diffusion-weighted imaging

Clinical Parameter	DWI findings	Mean MD (±SD) in lesions	p-value
Disease severity	Increased MD in lesions	0.85 (±0.12) mm²/s	<0.001
Lesion burden	Correlation with MD values	0.91 (±0.08) mm²/s	0.003
Cognitive function	Association with MD alterations	0.88 (±0.09) mm²/s	0.012
Treatment response	Predictive value of MD changes	0.83 (±0.11) mm²/s	0.025

The relationship between diffusion tensor imaging (DTI) results and clinical characteristics in MS patients is shown in Table 4. The significance of the observed relationships is shown by the matching p-values that correlate to the mean FA values in the lesions. The findings show that FA values and disease severity are correlated (mean FA=0.32 ± 0.05, p<0.001) and that the presence of lesions with lower FA values is associated with a higher lesion load (mean FA=0.29 ± 0.04, p=0.002). Moreover, FA changes serve as a possible treatment marker (mean FA=0.34 ± 0.07, p=0.019) and are linked to cognitive performance (mean FA=0.31 ± 0.06, p=0.008). These results highlight the clinical use of DTI in evaluating MS patients' lesion load, cognitive function, disease severity, and responsiveness to therapy.

**Table 4:** Association Between DTI Findings and Clinical Parameters P-value of <0.05 is considered significant FA - fractional anisotropy; DTI - diffusion tensor imaging

Clinical parameter	DTI findings	Mean FA (±SD) in lesions	p-value
Disease severity	Correlation with FA values	0.32 (±0.05)	<0.001
Lesion burden	Reduced FA in lesions	0.29 (±0.04)	0.002
Cognitive function	FA alterations and cognition	0.31 (±0.06)	0.008
Treatment response	FA changes as treatment markers	0.34 (±0.07)	0.019

The relationship between MTI results and clinical indicators in MS patients is shown in Table 5. Values for the mean magnetization transfer ratio (MTR) in lesions are given with matching p-values that show how significant the correlations that have been detected are. The findings show that MTR values and disease severity are correlated (mean MTR=0.18 ± 0.03, p<0.001), and that MTR variations are related to the amount of lesions (mean MTR=0.16 ± 0.02, p=0.001). Furthermore, MTR variations have a prognostic value for treatment response (mean MTR = 0.19 ± 0.03, p=0.014) and are linked to cognitive performance (mean MTR=0.17 ± 0.04, p=0.006). These results highlight the clinical use of MTI in evaluating MS patients' lesion load, cognitive function, disease severity, and responsiveness to therapy.

**Table 5:** Association between MTI findings and clinical parameters P-value of <0.05 is considered significant MTI - magnetization transfer imaging; MTR - magnetization transfer ratio

Clinical parameter	MTI finding	Mean MTR (±SD) in lesions	p-value
Disease severity	MTR correlation with severity	0.18 (±0.03)	<0.001
Lesion burden	MTR changes in lesion load	0.16 (±0.02)	0.001
Cognitive function	MTR alterations and cognition	0.17 (±0.04)	0.006
Treatment response	Predictive value of MTR changes	0.19 (±0.03)	0.014

The relationship between clinical characteristics and functional magnetic resonance imaging (fMRI) results in MS patients is shown in Table 6. The significance of the observed relationships is shown by the associated p-values that correlate to the mean BOLD signal levels in the cognitive processing areas. The findings show a strong relationship (mean BOLD signal=0.82 ± 0.06, p<0.001) between the illness severity and the BOLD signal. Furthermore, BOLD signal modifications show a predictive value for treatment response (mean BOLD signal=0.84 ± 0.05, p=0.017) and are linked to cognitive performance (mean BOLD signal=0.80 ± 0.08, p=0.005). These results demonstrate the therapeutic usefulness of fMRI in assessing cognitive function, the severity of the illness, and the effectiveness of therapy in MS patients.

**Table 6:** Association Between fMRI Findings and Clinical Parameters P-value of <0.05 is considered significant fMRI - functional magnetic resonance imaging; BOLD - blood oxygen level-dependent

Clinical parameter	fMRI finding	Mean BOLD signal (±SD)	p-value
Disease severity	BOLD signal correlation with severity	0.82 (±0.06)	<0.001
Cognitive function	BOLD signal changes and cognition	0.80 (±0.08)	0.005
Treatment response	Predictive value of fMRI alterations	0.84 (±0.05)	0.017

The relationships between advanced MRI results and clinical indicators in MS patients are presented in Table 7. In DWI, there are noteworthy associations between mean diffusivity (MD) values and the following: lesion burden (mean MD=0.91 ± 0.08 mm²/s, p=0.003), cognitive function (mean MD=0.88 ± 0.09 mm²/s, p=0.012), disease severity (mean MD=1.25 ± 0.15 mm²/s, p<0.001), and treatment response (mean MD=0.83 ± 0.11 mm²/s, p=0.025). Comparably, mean FA values in DTI exhibit significant correlations with lesion load (mean FA=0.29 ± 0.04, p=0.002), cognitive function (mean FA=0.31 ± 0.06, p=0.008), treatment response (mean FA=0.34 ± 0.07, p=0.019), and disease severity (mean FA=0.32 ± 0.05, p<0.001). These results highlight the clinical use of DWI and DTI in assessing MS patients' disease severity, lesion load, cognitive function, and responsiveness to therapy.

**Table 7:** Association between advanced MRI findings and clinical parameters P-value of <0.05 is considered significant DWI - diffusion-weighted imaging; DTI - diffusion tensor imaging; MTI - magnetization transfer imaging; fMRI - functional magnetic resonance imaging

MRI modality	Clinical parameter	Mean value (± SD)	p-value
DWI	Disease severity	1.25 ± 0.15 mm²/s	<0.001
	Lesion burden	0.91 ± 0.08 mm²/s	0.003
	Cognitive function	0.88 ± 0.09 mm²/s	0.012
	Treatment response	0.83 ± 0.11 mm²/s	0.025
DTI	Disease severity	0.32 ± 0.05	<0.001
	Lesion burden	0.29 ± 0.04	0.002
	Cognitive function	0.31 ± 0.06	0.008
	Treatment response	0.34 ± 0.07	0.019
MTI	Disease severity	0.18 ± 0.03	<0.001
	Lesion burden	0.16 ± 0.02	0.001
	Cognitive function	0.17 ± 0.04	0.006
	Treatment response	0.19 ± 0.03	0.014
fMRI	Disease severity	0.82 ± 0.06	<0.001
	Cognitive function	0.80 ± 0.08	0.005
	Treatment response	0.84 ± 0.05	0.017

## Discussion

Our investigation into the role of advanced MRI sequences in MS revealed compelling findings across various modalities. Our investigation showed a considerable rise in mean diffusivity (MD) inside white matter lesions, starting with DWI and reaching a mean value of 1.25 ± 0.15 mm²/s. This increase in MD was highly linked with lesion load (mean MD=0.91 ± 0.08 mm²/s, p=0.003), cognitive function (mean MD = 0.88 ± 0.09 mm²/s, p=0.012), therapy response (mean MD=0.83 ± 0.11 mm²/s, p=0.025), and disease severity (mean MD = 0.85 ± 0.12 mm²/s, p<0.001). Increased MD values were linked to more severe illness symptoms, pointing to a possible link between MD changes and the advancement of multiple sclerosis. Moreover, DWI-derived MD alterations showed potential as biomarkers for tracking MS patients' response to treatment. Consistent results were found when comparing DWI-derived MD values with prior research studies in relation to disease severity, lesion load, cognitive function, and responsiveness to therapy [19,20]. Our findings are supported by these investigations, which highlight the clinical value of DWI in assessing MS pathology and treatment effects.

Likewise, our DTI study revealed a decrease in FA with a mean FA value of 0.40 ± 0.08 inside white matter, which seems normal. The disease severity (mean FA=0.32 ± 0.05, p<0.001), lesion burden (mean FA=0.29 ± 0.04, p=0.002), cognitive function (mean FA=0.31 ± 0.06, p=0.008), and treatment response (mean FA=0.34 ± 0.07, p=0.019) were all substantially correlated with this decline in FA values. The connections that have been identified highlight the possibility of using FA variations generated from DTI as predictive markers for the advancement of MS and cognitive decline. Furthermore, FA alterations could be useful indicators for tracking the effectiveness of treatments and directing therapeutic approaches in MS patients. Similar relationships between DTI-derived FA values and MS patients' treatment response, cognitive function, and disease severity were found when comparing these results to earlier research [21, 22]. These consistent results bolster the therapeutic usefulness of DTI in the therapy of multiple sclerosis and confirm the robustness of our findings.

Moving on to MTI, our research found that MS lesions had a lower magnetization transfer ratio (MTR), with a mean value of 0.15 ± 0.03. The MTR drop showed noteworthy associations with lesion load (mean MTR=0.16 ± 0.02, p=0.001), cognitive function (mean MTR=0.17 ± 0.04, p=0.006), treatment response (mean MTR=0.19 ± 0.03, p=0.014), and disease severity (mean MTR=0.18 ± 0.03, p<0.001). The correlations that have been discovered underscore the potential of MTI as an accurate means of evaluating myelin integrity and disease activity in multiple sclerosis. Clinicians may be able to better understand the course of the illness and the effectiveness of therapy by measuring changes in MTR, which may help them make more educated treatment choices for MS patients. Comparing our results on the effectiveness of MTI in assessing MS severity and treatment response with previous research [23, 24]. The clinical significance of MTR changes as indicators for disease activity and treatment results in MS patients is further supported by these investigations.

Moreover, our fMRI study showed that cognitive processing areas had changed BOLD signals, with a mean BOLD signal value of 0.75 ± 0.10. BOLD signal alterations showed noteworthy correlations with illness intensity (average BOLD signal = 0.82 ± 0.06, p < 0.001), mental capacity (average BOLD signal=0.80 ± 0.08, p=0.005), and reaction to therapy (average BOLD signal = 0.84 ± 0.05, p = 0.017). The discovered functional changes provide insight into the central nervous system's compensatory networks and adaptive processes in response to damage caused by multiple sclerosis. Furthermore, fMRI results may help predict cognitive outcomes and treatment responses in MS patients, allowing for the development of individualized management plans. Our findings on the relationship between fMRI-derived BOLD signals and MS patients' disease severity, cognitive function, and response to therapy were confirmed by comparison with prior research [25]. These findings provide credence to fMRI's potential as a useful method for evaluating functional brain alterations and forecasting clinical outcomes in multiple sclerosis.

Limitation

Acknowledging the retrospective nature of the study, which relies on existing patient records, introduces potential biases such as selection bias and variability in data completeness across medical centers. Despite these constraints, the study offers valuable insights into the associations between advanced MRI biomarkers and clinical factors in multiple sclerosis. Future research employing prospective study designs, such as cohort studies or randomized controlled trials with longitudinal follow-ups, could provide deeper knowledge and stronger evidence. These approaches would offer a more comprehensive understanding of the complex relationship between advanced MRI findings and clinical outcomes in the progression and treatment response of multiple sclerosis.

## Conclusions

Strong relationships were found between imaging biomarkers and clinical measures, including disease severity, lesion load, cognitive function, and responsiveness to therapy, utilizing DWI, DTI, MTI, and fMRI. This research contributes to understanding the pathophysiology of MS and provides valuable insights for personalized treatment strategies. The use of advanced MRI methods enables physicians to optimize patient care, predict treatment outcomes, and monitor disease progression, thereby enhancing the quality of life for individuals with multiple sclerosis. However, further investigation and validation are required to fully realize the clinical potential of advanced MRI in MS therapy.
